# The concordance of swelling/tenderness with ultrasound-detected inflammatory lesions in patients with psoriatic arthritis

**DOI:** 10.3389/fimmu.2025.1562996

**Published:** 2025-05-30

**Authors:** Xiaoying Sun, Yan Geng, Zhibo Song, Xuerong Deng, Xinyi Hu, Yu Wang, Xiaohui Zhang, Juan Zhao, Guangtao Li, Lanlan Ji, Wenhui Xie, Hong Huang, Zhuoli Zhang

**Affiliations:** Department of Rheumatology and Clinical Immunology, Peking University First Hospital, Beijing, China

**Keywords:** arthritis, psoriatic, ultrasonography, inflammation, physical examination

## Abstract

**Background:**

Psoriatic arthritis (PsA) is a complex and varied inflammatory condition that can cause arthritis, enthesitis, dactylitis, and spondylitis. In recent years, ultrasound (US) imaging has emerged as a valuable adjunct to physical examination (PE) in the assessment of PsA. This study aims to assess the concordance between clinical manifestations of swelling/tenderness and US-detected inflammatory lesions in the wrists and hands of patients with PsA.

**Methods:**

The study utilized the PKUPsA cohort and included both clinical and US evaluations of 30 joints per PsA patient, encompassing bilateral wrists, proximal interphalangeal (PIP), metacarpophalangeal (MCP), and distal interphalangeal (DIP) joints. Clinical assessments included the detection of tenderness or swelling, while US evaluations identified synovitis, tenosynovitis/paratenonitis, enthesitis, and soft tissue inflammation. Cohen’s kappa (κ) statistic was employed to measure the concordance between clinical and sonographic findings.

**Results:**

A total of 188 patients with PsA were included in the study. US-detected inflammatory lesions were more common in swollen joints than tender joints (50.6% vs. 40.3%, p<0.01). The overall concordance between clinical findings and US-detected inflammatory lesions was found to be moderate (κ=0.448, p<0.01). Joint swelling showed a higher level of concordance with US-detected inflammation (κ=0.497, p<0.01) than tenderness (κ=0.406, p<0.01). In the MCPs and wrists, synovitis exhibited a higher concordance with PE than tenosynovitis/paratenonitis. In contrast, in most PIP joints, US-detected tenosynovitis/paratenonitis aligned more closely with PE than synovitis. In DIP joints, enthesitis showed a greater concordance with PE than both synovitis and tenosynovitis/paratenonitis.

**Conclusions:**

Ultrasound-detected inflammatory lesions in PsA patients showed a moderate level of concordance with PE in PsA patients, but significant discrepancies were observed across different joints and lesion types. These findings highlight the importance of incorporating US into the routine management for a more comprehensive understanding of PsA.

## 1 Introduction

Psoriatic arthritis (PsA) is a chronic inflammatory disease characterized by a diverse range of musculoskeletal manifestations, including arthritis, enthesitis, dactylitis and spondylitis, in addition to psoriatic skin and nail lesions (1). Traditionally, the clinical assessment of joints has relied on physical examination (PE) to determine the presence or absence of tenderness and/or swelling. However, accurate assessment of enthesitis and dactylitis, the key features of PsA, can often be challenging (2). In recent years, imaging techniques have gained prominence in the differential diagnosis and monitoring of treatment response in PsA (3). The European League Against Rheumatism (EULAR) has recommended the use of imaging modalities such as magnetic resonance imaging and ultrasound (US), for the diagnosis and management of spondyloarthritis (SpA) (4). These imaging techniques are valuable for monitoring disease activity and evaluating structural changes in peripheral SpA. In particular, US has been shown to be effective in detecting inflammation in tendons, entheses, and synovium, offering better accuracy and sensitivity compared to PE. By complementing clinical assessment, US enhances differential diagnosis, aids in patient stratification, and informs therapeutic strategies within a treat-to-target (T2T) framework (5, 6).

The concordance between clinical and ultrasound findings in rheumatoid arthritis (RA) has been extensively explored, with results showing weak to moderate agreement (7, 8). In contrast, the investigation of this agreement in PsA has been less extensive. The small joints of hands are frequently involved in both RA and PsA, but there are distinct differences in the types of lesions observed on US between these two conditions. US of the hand has been shown to offer additional value compared to PE and laboratory tests in the differential diagnosis of PsA (5, 6). The efficiency of ultrasound in detecting subclinical inflammation in PsA patients with arthritis involving hand joints has been proven (9). Furthermore, US scans of the wrists and hands are easy to perform, effective, and reproducible in clinical practice.

The primary objective of this study was to evaluate the concordance between clinical signs (swelling/tenderness) and ultrasound-detected inflammatory lesions in the wrists and hands of patients with PsA. Secondary objectives were to compare concordance patterns between different joint types and lesion types (synovitis, tenosynovitis/paratenonitis, enthesitis and soft tissue inflammation) and to explore associations between ultrasound findings and clinical measures of disease activity.

## 2 Methods

### 2.1 Patients

This cross-sectional study utilized data from the Peking University First Hospital PsA (PKUPsA) cohort (a real-world cohort established in 2010) and consecutively enrolled patients from January 2019 to June 2024 who met all of the following key inclusion criteria: definite clinical diagnosis of PsA meeting the Classification Criteria for Psoriatic Arthritis (CASPAR), age ≥18 years, presence of ≥1 clinically active joint (tender or swollen) among 30 assessed joints (bilateral wrists, metacarpophalangeal [MCP] 1-5, proximal interphalangeal [PIP] 1-5, and distal interphalangeal [DIP] 2-5) and completion of comprehensive bilateral wrist and hand ultrasonography (10). Patients were excluded if they had comorbid inflammatory arthritis (RA, gout, etc.), a history of trauma or joint replacement surgery involving the wrists/hands, or significant joint deformity/mutilation. The study was approved by the Ethical Committee of Peking University First Hospital (No.2019-267), and the informed consent was obtained from each participant.

### 2.2 Data collection

In this study, a comprehensive collection of demographics, clinical, and laboratory data were collected. The medication regimens, including conventional synthetic disease-modifying anti-rheumatic drugs (csDMARDs) such as methotrexate, leflunomide, and sulfasalazine, as well as glucocorticoids, biologic DMARDs (bDMARDs) encompassing tumor necrosis factor (TNF) inhibitors, interleukin-17 (IL-17) inhibitors, interleukin-23 (IL-23) inhibitors, targeted synthetic DMARDs (tsDMARDs) including Janus kinase (JAK) inhibitors and phosphodiesterase 4 (PDE4) inhibitors, and non-steroidal anti-inflammatory drugs (NSAIDs), were documented.

Peripheral joint assessment included tenderness based on 68 joints and swelling based on 66 joints. This assessment was performed independently by a rheumatologist (Dr. Zhibo Song) who was blinded to both clinical and ultrasound data to ensure objectivity. Additionally, dactylitis of the fingers and toes was recorded. Patient’s global assessment of disease activity (PGA), patient’s pain assessment (Ptpain) and evaluator’s global assessment of disease activity (EGA) were measured on a Visual Analogue Scale ranging from 0 to 100 mm. The leeds enthesitis index (LEI) was also recorded. Skin lesions and the Psoriasis Area and Severity Index (PASI) for psoriasis vulgaris were evaluated by a trained rheumatologist (11). The minimal disease activity (MDA), Health Assessment Questionnaire (HAQ) and a quality-of-life measure (Psoriatic Arthritis Quality of Life, PsAQoL) were also documented. MDA was defined by fulfilling five out of seven of the following criteria: tender joint count (TJC)≤1, swollen joint count (SJC) ≤1, PASI ≤1, Ptpain≤15 mm, PGA≤20 mm and HAQ≤0.5; enthesitis score (out of clinical MASEI+E) ≤1 (12).

Laboratory measurements included levels of C-reactive protein (CRP), erythrocyte sedimentation rate (ESR), anti-cyclic citrullinated peptide antibody (CCP) and rheumatoid factor (RF), along with determination of human leukocyte antigen B27 (HLA-B27) status. Disease activity was assessed by using the Disease Activity Index for Psoriatic Arthritis (DAPSA) score (13).

### 2.3 US assessment

The US evaluation in this study was conducted by two highly experienced sonographers. The intra-observer reliability of their evaluations was high, with coefficients of 0.885 (95% CI 0.797-0.935) and 0.794 (95% CI 0.648-0.879), respectively. The inter-observer reliability between the two operators was excellent, with a coefficient of 0.986 (95% CI 0.981-0.990), indicating a high level of consistency in their assessments. US examinations were performed on 30 joints (bilateral wrists, MCP1-5, PIP1-5, DIP2-5). The ultrasound protocol included transverse and longitudinal scans of the joints. A GE machine (LOGIQ E9 USA) with linear ML 15–6 MHz or small-footprint linear array 18–8 MHz transducers was used. Power Doppler (PD) settings included a pulse repetition frequency (PRF) of 500–1000 Hz, low wall filter, and Doppler gain, which were adjusted to produce the higher sensitivity but avoid random noise visualization. PE and US assessment of the joints were performed blindly on the same visiting day to ensure that the assessments were independent and not influenced by the results of the other.

Inflammatory lesions detected by US were defined as the presence of one or more of the following, synovitis, tenosynovitis/paratenonitis, enthesitis, or soft tissue inflammation. Scanning and interpretation of synovitis, tenosynovitis, enthesitis, osteophytes, and bone erosions, were based on the Outcome Measures in Rheumatology Clinical Trials (OMERACT) definitions (14–16). A semi-quantitative scoring system proposed by Szkudlarek et al. was used to grade the synovial hypertrophy/synovitis (17). Synovitis was defined as grey scale (GS) score ≥2 and/or PD score ≥1, recognizing that low-level GS changes can be seen in the wrist and hands of healthy subjects (18). Tenosynovitis was investigated by both palmar and dorsal scans of the wrists and palmar scans of MCP, PIP, and DIP joints. Extensor paratenonitis (peritendon extensor inflammation), defined as hypoechoic thickening (with or without power Doppler signal) surrounding extensor tendons, was evaluated by systematic dorsal scanning of the MCP, PIP, and DIP joints (19, 20). Tenosynovitis/paratenonitis was dichotomously scored (0=absent, 1=present) based on GS and/or PD signals, with positivity recorded when either lesion type was detected in each evaluated joint. Enthesitis was assessed at the extensor and flexor tendon insertions at the distal phalanges of the bilateral 2nd-5th DIP joints and PIP1 joints, defined by the presence of hypoechogenicity, thickening, and/or PD signals (21, 22). Soft tissue inflammation was defined as soft tissue edema accompanied by PD signals. Both enthesitis and soft tissue inflammation were dichotomously scored (0=absent, 1=present). Osteophytes and bone erosions were dichotomously scored (0=absent, 1=present) for each joint region (wrists, MCPs, PIPs or DIPs), with patient-level positivity rates calculated for each region (defined as positive when ≥1 joint in the region exhibited pathological changes).

**Figure 1:**
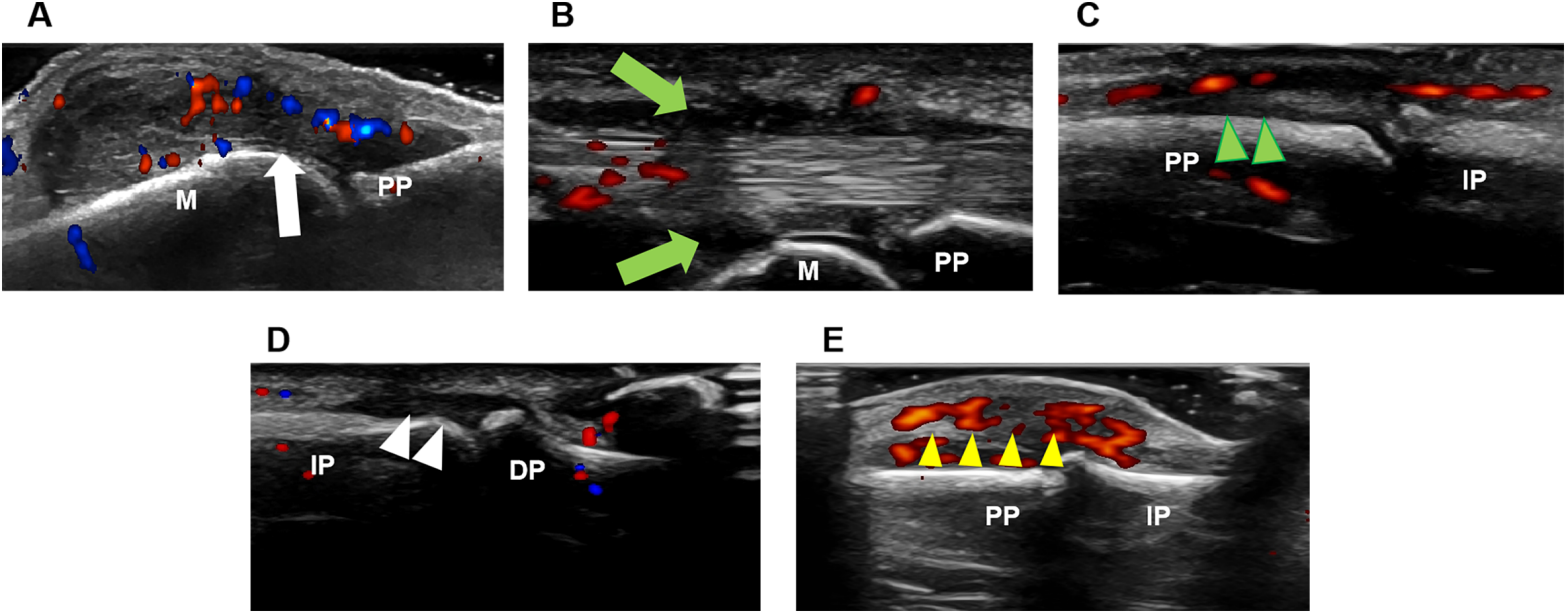
Ultrasound imaging of diverse inflammatory lesions in the hand. **(A)** Synovitis (white arrow). **(B)** Tenosynovitis (green arrow). **(C)** Paratenonitis (green arrowhead). **(D)** Enthesitis (white arrowhead). **(E)** Soft tissue inflammation (yellow arrowhead). DP, distal phalanx; IP, intermediate phalanx; M, metacarpal bone; PP, proximal phalanx.

A comprehensive global ultrasound sum score (range 0-170) was developed to quantify the inflammatory burden in the wrists and hands of PsA patients. The composite score incorporated seven parameters, with each parameter scored dichotomously (0 = absent, 1 = present) at every assessed joint: synovial hypertrophy (GS≥2) (scored 0-30), synovitis (PD≥1) (scored 0-30), GS-defined tenosynovitis/paratenonitis (scored 0-30), PD-positive tenosynovitis/paratenonitis (scored 0-30), GS-defined enthesitis (scored 0-10), PD-positive enthesitis (scored 0-10) and soft tissue inflammation (scored 0-30). Ultrasound images of diverse inflammatory lesions in the hand were shown in **Figure 1**.

### 2.4 Statistical analysis

Statistical analysis for this study was performed using SPSS version 27.0. Descriptive analyses were conducted for continuous variables, presented as mean and standard deviation (SD) if normally distributed, or as median and interquartile range (IQR) if not. Independent t-tests were used to compare continuous variables between groups if the data met normality assumptions, and Wilcoxon signed-rank tests were used if these assumptions were not met. Categorical or dichotomous variables were reported as frequencies and percentages, and comparisons between groups were made using the χ2 test. Absolute agreement and Cohen’s kappa (κ) statistics were calculated to assess the concordance between clinical and sonographic findings. The interpretation of κ coefficients was categorized as follows: 0-0.20 for poor, 0.20-0.40 for fair, 0.40-0.60 for moderate, 0.60-0.80 for good and 0.80-1.00 for excellent agreement. These categories provide a clear framework for interpreting the strength of agreement between the two sets of findings. It’s important to note that kappa values can be influenced by the prevalence of categories in the data, and even very good agreements can result in low kappa values if the data are skewed. Correlations were analyzed using Spearman’s rank correlation test. P values <0.05 were set as the threshold for statistical significance.

## 3 Results

### 3.1 Demographics and clinical characteristics of patients

A total of 188 patients with PsA were enrolled in this study. The demographic and clinical characteristics are summarized in **Table 1**. The median (IQR) age was 41.8 (31.3, 52.0) years, with 44.7% female patients. The median (IQR) disease duration for arthritis was 36 (12, 96) months, whereas the median (IQR) duration of psoriasis was 168 (84, 272) months. Skin lesions were observed in 176 (93.6%) patients, and 102 (54.3%) patients had nail lesions. The median (IQR) PASI score was 2.4 (0.3, 5.9). There were 59 (31.4%) patients with current dactylitis. The median (IQR) TJC and SJC were 3 (2, 8) and 3 (2, 6), respectively. The median (IQR) Ptpain, PGA and EGA score were 30 (15, 50), 30 (20, 50), and 30 (18, 40), respectively. There were 25 (13.3%) patients who achieved MDA. As for the types of arthritis, DIP joint disease was noted in 10.1% of the patients, oligoarthritis in 35.1%, polyarthritis in 53.2%, mutilans subtype in 6.9%, and axial disease in 10.6%. The median (IQR) levels of ESR were 12 (6, 27) mm/h and CRP 4.9 (1.8, 12.5) mg/L. Positive RF, anti-CCP, and HLA-B27 were present in 5.1%, 3.2%, and 6.6% of patients, respectively. The mean (SD) DAPSA score was 19.8 (14.2), indicating moderate disease activity.

**Table 1:** Demographics and clinical characteristics of 188 PsA patients.

Characteristics and outcomes	Values
Demographics and clinical features	
Age, years, median (IQR)	41.8 (31.3, 52.0)
Female, n (%)	84 (44.7)
Family history of PsO/PsA, n (%)	59 (31.4)
Smoker, n (%)*	60 (31.9)
BMI, kg/m², median (IQR)	24.4 (22.3, 26.6)
Arthritis duration, months, median (IQR)	36 (12, 96)
Psoriasis duration, months, median (IQR)	168 (84, 272)
Skin lesions, n (%)*	176 (93.6)
PASI (0–72), median (IQR)	2.4 (0.3, 5.9)
Nail lesions, n (%)*	102 (54.3)
Patients with current dactylitis, n (%)	59 (31.4)
LEI in patients with enthesitis (0–6), median (IQR)	0 (0, 0)
TJC (0–68), median (IQR)	3 (2, 8)
SJC (0–66), median (IQR)	3 (2, 6)
Ptpain (0-100mm), median (IQR)	30 (15, 50)
PGA (0-100mm), median (IQR)	30 (20, 50)
EGA (0-100mm), median (IQR)	30 (18, 40)
Phenotypes of PsA	
DIP joint disease, n (%)	19 (10.1)
Oligoarthritis, n (%)	66 (35.1)
Polyarthritis, n (%)	100 (53.2)
Mutilans, n (%)	13 (6.9)
Axial disease, n (%)	20 (10.6)
ESR, mm/h, median (IQR)	12 (6, 27)
CRP, mg/L, median (IQR)	4.9 (1.8, 12.5)
Positive RF, n (%)^*^	7 (5.1)
Positive anti-CCP, n (%)^*^	4 (3.2)
Positive HLA-B27, n (%)^*^	8 (6.6)
HAQ (0–3), median (IQR)	0.05 (0, 0.28)
PsAQoL (0–20), median (IQR)	1 (0, 4)
DAPSA, mean (SD)	19.8 (14.2)
MDA attainment, n (%)	25 (13.3)
Medication regimens	
Current use of prednisolone, n (%)	1 (0.5)
Current use of NSAIDs, n (%)	14 (7.4)
Current use of csDMARDs, n (%)	87 (46.3)
Methotrexate, n (%)	82 (43.6)
Sulfasalazine, n (%)	13 (6.9)
Leflunomide, n (%)	10 (5.3)
Current use of tsDMARDs or bDMARDs, n (%)	30 (16.0)
IL-17i, n (%)	11 (5.9)
TNFi, n (%)	10 (5.3)
JAKi, n (%)	8 (4.3)
PDE4i, n (%)	2 (1.1)
IL-23i, n (%)	1 (0.5)

Values are presented as n (%) for binary variables and mean ± SD or median (IQR) for continuous variables. ^*^: Past and/or currently involved. anti-CCP, anti-cyclic citrullinated peptide antibody; bDMARDs, biological disease-modifying anti-rheumatic drugs; BMI, body mass index; CRP, C-reactive protein; csDMARDs, conventional synthetic disease-modifying anti-rheumatic drugs; DAPSA, disease activity index for psoriatic arthritis; DIP, distal interphalangeal; EGA, evaluator’s global assessment of disease activity; ESR, erythrocyte sedimentation rate; HAQ, Health Assessment Questionnaire; HLA-B27, human leukocyte antigen B27; IL-17i, interleukin-17 inhibitors; IL-23i, interleukin-23 inhibitors; IQR, interquartile range; JAKi, Janus kinase inhibitors; LEI, Leeds enthesitis index; MDA, minimal disease activity; NSAIDs, non-steroidal anti-inflammatory drugs; PASI, psoriasis area and severity index; PDE4i, phosphodiesterase 4 inhibitors; PGA, patient’s global assessment of disease activity; PsA, psoriatic arthritis; PsAQoL, psoriatic arthritis quality of life; PsO, psoriasis; Ptpain, patients’ pain assessment; RF, rheumatoid factor; SD, standard deviation; SJC, swollen joint count; TJC, tender joint count; TNFi, tumour necrosis factor inhibitors; tsDMARDs, targeted synthetic disease-modifying anti-rheumatic drugs.

In this cohort, 88 (46.8%) patients were (DMARD)-naïve. csDMARDs were used by 46.3% of the patients, with methotrexate being the most commonly prescribed agent (43.6%). 30 (16.0%) patients were receiving b/tsDMARDs, with IL-17i (5.9%) and TNFi (5.3%) being the most commonly used agents. Only 0.5% of patients were on concurrent prednisone with daily dose 5 mg, and 7.4% were treated with NSAIDs.

### 3.2 Physical examination and ultrasound-detected inflammatory lesions in wrist and finger joints

A total of 5,640 joints were assessed. Of these, 10.3% (n=581) were found to be swollen, and 12.1% (n=685) were tender upon clinical examination (**Table 2**). Ultrasound evaluation revealed GS synovial hypertrophy in 6.4% (n=362) of the joints, PD synovitis in 3.2% (n=183), tenosynovitis/paratenonitis on GS in 3.5% (n=196) and on PD in 2.3% (n=127). GS identified enthesitis was identified in 5.2% (n=98) of joints, with PD signal detected in 2.9% (n=54) of joints. Soft tissue inflammation was present in only 0.1% (n=4).

**Table 2:** Frequencies of tenderness/swelling and ultrasound-detected inflammatory lesions.

Joints	PE		US				
	Tenderness	Swelling	GS synovial hypertrophy	PD synovitis	Tenosynovitis/ paratenonitis	Enthesitis	Ultrasound-detected inflammatory lesions
MCP1	12.5%	9.0%	12.0%	6.6%	1.1%	NA	9.3%
MCP2	10.1%	10.9%	9.6%	7.4%	4.8%	NA	10.9%
MCP3	5.05%	5.6%	4.8%	3.7%	4.5%	NA	8.5%
MCP4	6.9%	3.2%	6.9%	4.5%	1.6%	NA	6.6%
MCP5	3.5%	4.5%	6.1%	3.2%	2.1%	NA	6.1%
PIP1	17.3%	16.0%	6.4%	5.3%	2.4%	2.4%	9.3%
PIP2	16.5%	12.2%	4.5%	2.7%	6.1%	NA	9.3%
PIP3	18.1%	13.6%	3.7%	2.4%	6.4%	NA	9.6%
PIP4	15.4%	11.2%	4.0%	2.7%	5.9%	NA	8.2%
PIP5	12.5%	10.1%	1.6%	1.1%	4.0%	NA	5.3%
DIP2	16.0%	15.4%	2.4%	0.5%	2.7%	8.2%	11.4%
DIP3	10.1%	10.4%	2.7%	0.8%	1.3%	5.9%	8.0%
DIP4	10.4%	10.4%	1.9%	1.3%	1.6%	5.1%	7.4%
DIP5	10.1%	9.8%	1.9%	0.3%	1.1%	4.5%	5.6%
Wrist	17.8%	12.2%	27.9%	6.1%	6.6%	NA	16.8%
At joint level	12.1%	10.3%	6.4%	3.2%	3.5%	5.2%	8.8%
At patient level	92.0%	89.9%	64.9%	40.4%	50.5%	25.0%	78.2%

DIP, distal interphalangeal; GS, grey scale; MCP, metacarpophalangeal; NA, not applicable; PD, power Doppler; PE, physical examination; PIP, proximal interphalangeal; US, ultrasound. Ultrasound-detected inflammatory lesions: GS≥2 or PD≥1 for synovitis, tenosynovitis/paratenonitis, enthesitis, or soft tissue inflammation.

**Figure 2:**
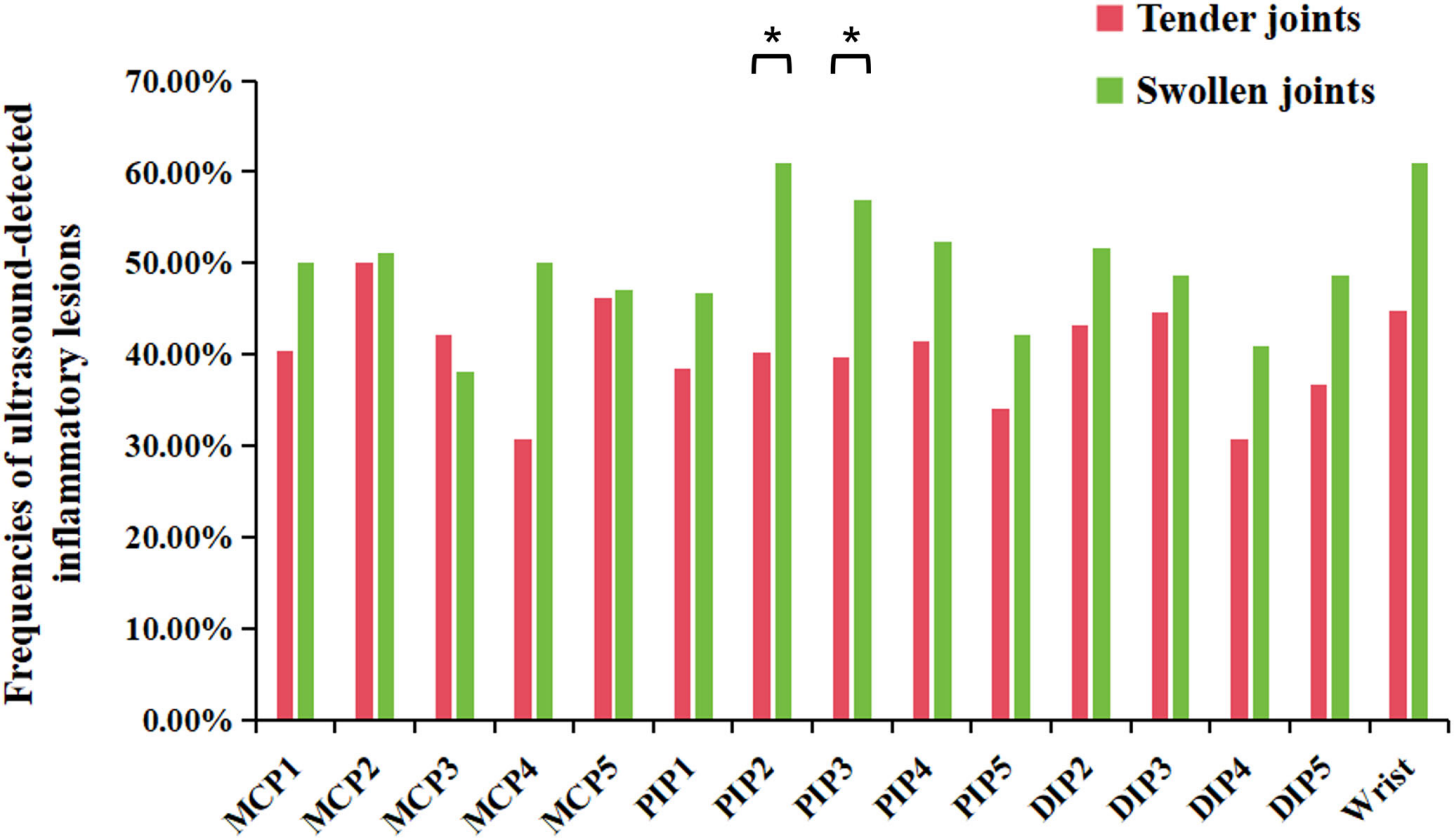
Frequencies of ultrasound-detected inflammatory lesions in tender or swollen joints in wrist and finger joints. DIP, distal interphalangeal; MCP, metacarpophalangeal; PIP, proximal interphalangeal. *P-value<0.05.

Clinical examination showed that tenderness/swelling was most commonly found in PIP1 joints and wrists, while US-detected joint inflammation was predominantly observed in the wrists. Enthesitis, as detected by ultrasound, was most common in DIP2 joints. Notably, US-detected inflammatory lesions were more common in swollen joints than in tender joints (50.6% vs. 40.3%, p<0.01). This trend was evident across most wrist and finger joints, although statistically significant differences were noted specifically in PIP2 and PIP3 joints (p<0.05) (**Figure 2**). There were more joints that were tender without swelling than joints that were swollen without tenderness (4.3% vs. 2.4%, p<0.01). The frequency of US-detected inflammatory lesions was significantly higher in swollen joints without tenderness than in tender joints without swelling (32.6% vs. 14.9%, p<0.01).

### 3.3 Concordance of tenderness/swelling with ultrasound-detected inflammatory lesions

The overall agreement between PE and ultrasound-detected inflammatory lesions was moderate, with a κ coefficient of 0.448 (p<0.01) (**Table 3**). Swelling showed a higher concordance with ultrasound-detected inflammation than tenderness (κ=0.497 vs. κ=0.406, p<0.01). The highest concordance was observed in the DIP2 joint (κ=0.522, p<0.01), followed by the DIP3 joint (κ=0.513, p<0.01). Conversely, the lowest concordance was observed in the MCP4 joint (κ=0.306, p<0.01), followed by the MCP3 joint (κ=0.359, p<0.01). In PIPs and some DIP joints, there were more joints that were tender or swollen without ultrasound-determined inflammatory lesions (PE+/US-) and fewer joints that were not tender or swollen but had ultrasound-determined inflammatory (PE-/US+) compared to MCP joints. Notably, the wrist exhibited a greater number of joints with subclinical inflammation (PE-/US+) compared to other joint regions.

**Table 3:** The concordance between physical examination and ultrasound-detected inflammatory lesions.

Joints	Number of joints				Agreement (%)	κ coefficient
	PE+/US+	PE+/US-	PE-/US+	PE-/US-		
MCP1	22	33	13	308	87.8%	0.423^**^
MCP2	24	28	17	307	88.0%	0.449^**^
MCP3	13	18	19	326	90.2%	0.359^**^
MCP4	10	21	15	330	90.4%	0.306^**^
MCP5	10	13	13	340	93.1%	0.398^**^
PIP1	29	49	6	292	85.4%	0.442^**^
PIP2	29	43	6	298	87.0%	0.476^**^
PIP3	31	44	5	296	87.0%	0.493^**^
PIP4	26	41	5	304	87.8%	0.471^**^
PIP5	18	38	2	318	89.4%	0.429^**^
DIP2	33	36	10	297	87.8%	0.522^**^
DIP3	21	24	9	322	91.2%	0.513^**^
DIP4	16	31	12	317	88.6%	0.368^**^
DIP5	18	27	3	328	92.0%	0.508^**^
Wrist	36	41	27	272	81.9%	0.405^**^
Total	336	487	162	4655	88.5%	0.448^**^

DIP, digital interphalangeal; MCP, metacarpophalangeal; PE+, presence of tenderness or swelling by physical examination; PIP, proximal interphalangeal; US+, GS≥2 or PD≥1 for synovitis, tenosynovitis/paratenonitis, enthesitis, or soft tissue inflammation.**, P-value<0.01.

**Figure 3:**
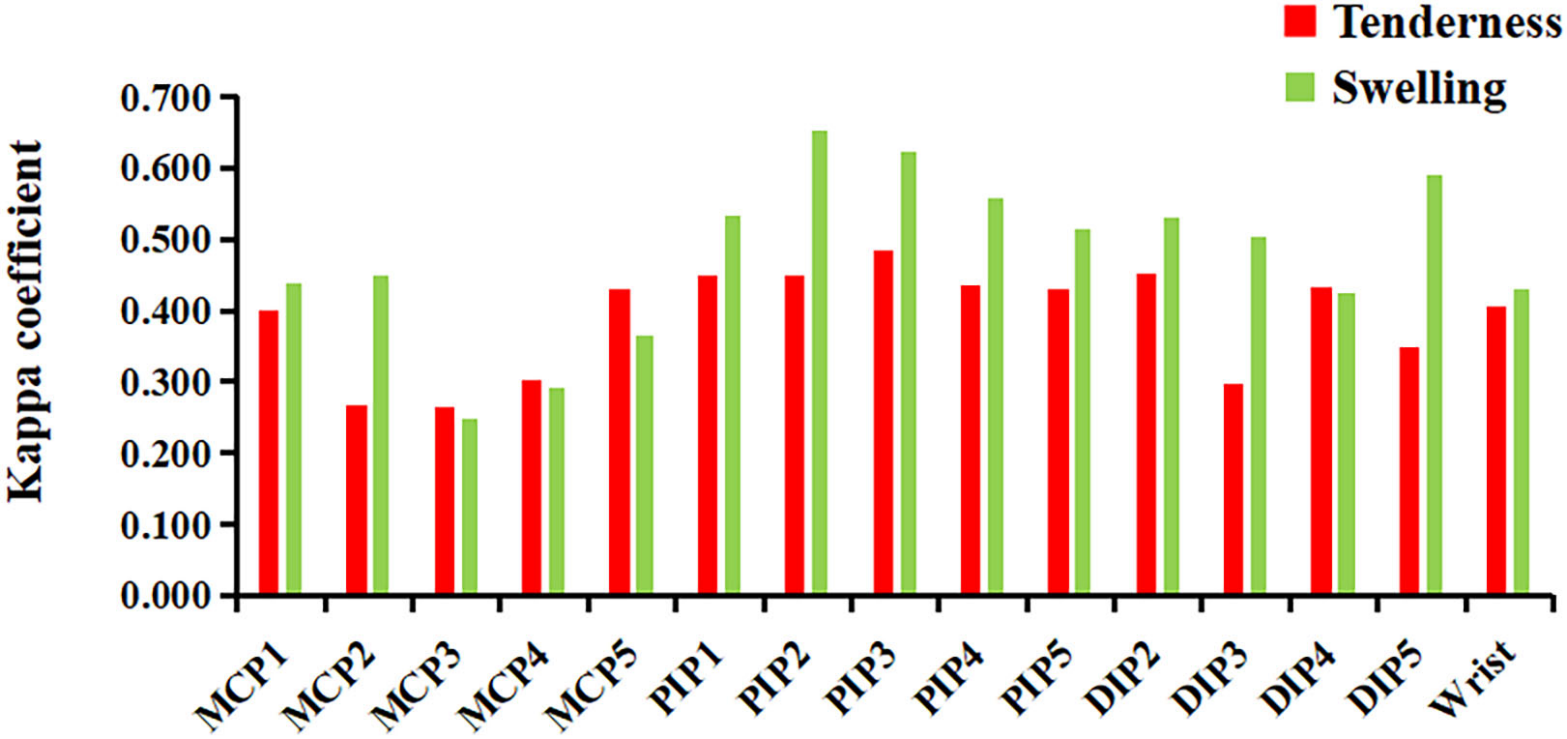
The concordance (κ coefficient) between joint tenderness/swelling and ultrasound-detected inflammatory lesions. DIP, distal interphalangeal; MCP, metacarpophalangeal; PIP, proximal interphalangeal. Ultrasound-detected inflammatory lesions: GS≥2 or PD≥1 for synovitis, tenosynovitis/paratenonitis, enthesitis, or soft tissue inflammation.

In most joints, swelling showed a higher κ coefficient with ultrasound-determined inflammation, ranging from 0.251 to 0.655 (p<0.01), compared to tenderness, which ranged from 0.264 to 0.484 (p<0.01). However, in the MCP3–5 joints, tenderness showed a higher κ coefficient with ultrasound-determined inflammation than swelling (**Figure 3**).

Enthesitis (κ=0.357, p<0.01) demonstrated a stronger concordance with tenderness/swelling compared to ultrasound synovitis (κ=0.269, p<0.01) and tenosynovitis/paratenonitis (κ=0.225, p<0.01) across all joints. In MCPs and wrists, synovitis had a higher concordance with PE than tenosynovitis/paratenonitis. Conversely, in PIP2–5 joints, ultrasound-detected tenosynovitis/paratenonitis had better concordance with PE than synovitis. In DIP joints, enthesitis showed greater concordance with PE than both synovitis and tenosynovitis/paratenonitis (**Table 4**).

**Table 4:** The concordance between physical examination and synovitis, tenosynovitis/paratenonitis, or enthesitis on ultrasound.

Joints	κ coefficient		
	Synovitis	Tenosynovitis/paratenonitis	Enthesitis
MCP1	0.387^**^	0.118^**^	NA
MCP2	0.411^**^	0.200^**^	NA
MCP3	0.256^**^	0.248^**^	NA
MCP4	0.258^**^	0.195^**^	NA
MCP5	0.314^**^	0.234^**^	NA
MCPs	0.346^**^	0.193^**^	NA
PIP1	0.340^**^	0.099^**^	0.147^**^
PIP2	0.267^**^	0.339^**^	NA
PIP3	0.251^**^	0.318^**^	NA
PIP4	0.257^**^	0.347^**^	NA
PIP5	0.136^**^	0.354^**^	NA
PIPs	0.260^**^	0.289^**^	NA
DIP2	0.107^**^	0.111^**^	0.473^**^
DIP3	0.245^**^	0.098^**^	0.401^**^
DIP4	0.126^**^	0.087^*^	0.314^**^
DIP5	0.132^**^	0.147^**^	0.482^**^
DIPs	0.148^**^	0.112^**^	0.425^**^
Wrist	0.345^**^	0.259^**^	NA
Total	0.269^**^	0.225^**^	0.357^**^

DIP, distal interphalangeal; DIPs, DIP joints; MCP, metacarpophalangeal; MCPs, MCP joints; NA, not applicable; PIP, proximal interphalangeal; PIPs, PIP joints. *P-value<0.05, **P-value<0.01.

In most joints of the bilateral hands and wrists, swelling demonstrated a higher κ coefficient compared to tenderness, with various lesions identified by ultrasound, such as synovitis, tenosynovitis/paratenonitis, and enthesitis (**Table 5**).

**Table 5:** The concordance between joint tenderness/swelling and ultrasound-detected inflammatory lesions.

Joints	κ coefficient					
	Synovitis		Tenosynovitis/paratenonitis		Enthesitis	
	Tenderness	Swelling	Tenderness	Swelling	Tenderness	Swelling
MCP1	0.359^**^	0.394^**^	0.140^**^	0.142^**^	NA	NA
MCP2	0.395^**^	0.403^**^	0.236^**^	0.256^**^	NA	NA
MCP3	0.191^**^	0.176^**^	0.242^**^	0.224^**^	NA	NA
MCP4	0.206^**^	0.337^**^	0.166^**^	0.205^**^	NA	NA
MCP5	0.237^**^	0.261^**^	0.266^**^	0.300^**^	NA	NA
MCPs	0.308^**^	0.339^**^	0.202^**^	0.228^**^	NA	NA
PIP1	0.307^**^	0.440^**^	0.097^**^	0.138^**^	0.182^**^	0.168^**^
PIP2	0.257^**^	0.415^**^	0.341^**^	0.479^**^	NA	NA
PIP3	0.227^**^	0.339^**^	0.304^**^	0.319^**^	NA	NA
PIP4	0.269^**^	0.328^**^	0.372^**^	0.458^**^	NA	NA
PIP5	0.126^**^	0.205^**^	0.348^**^	0.420^**^	NA	NA
PIPs	0.246^**^	0.361^**^	0.293^**^	0.380^**^	NA	NA
DIP2	0.126^**^	0.131^**^	0.102^**^	0.137^**^	0.364^**^	0.476^**^
DIP3	0.289^**^	0.282^**^	0.071^*^	0.116^**^	0.352^**^	0.380^**^
DIP4	0.154^**^	0.154^**^	0.063	0.109^**^	0.223^**^	0.371^**^
DIP5	0.159^**^	0.163^**^	0.126^**^	0.179^**^	0.399^**^	0.566^**^
DIPs	0.176^**^	0.178^**^	0.094^**^	0.137^**^	0.340^**^	0.451^**^
Wrist	0.295^**^	0.416^**^	0.230^**^	0.306^**^	NA	NA
Total	0.253^**^	0.314^**^	0.224^**^	0.276^**^	0.302^**^	0.389^**^

DIP, distal interphalangeal; DIPs, DIP joints; MCP, metacarpophalangeal; MCPs, MCP joints; NA, not applicable; PIP, proximal interphalangeal; PIPs, PIP joints. *P-value<0.05, **P-value<0.01.

### 3.4 Dactylitis and ultrasound findings

Of the 59 patients with clinical dactylitis, a total of 18 fingers were affected, representing 1.0% of the total fingers examined. Ultrasound detected inflammatory lesions in 12 of these 18 fingers, representing 66.7% of the affected fingers. Specifically, synovitis and tenosynovitis/paratenonitis each made up 66.7% of the detectable inflammatory lesions, while soft tissue inflammation was found in 8.3% of the cases.

### 3.5 Association between clinical findings and ultrasound scores

**Supplementary Table 1** shows the association between clinical findings and ultrasound sum score of wrists and hands. The global ultrasound sum score of wrists and hands demonstrated significantly stronger correlations with SJC (ρ=0.488, p<0.01), DAPSA (ρ=0.406, p<0.01), and CRP (ρ=0.392, p<0.01), compared to subjective clinical assessments (HAQ, Ptpain, PGA) (all p<0.01). While exhibiting no significant correlation with PASI (ρ=-0.098, p>0.05), suggesting different mechanisms between cutaneous and articular involvement.

### 3.6 Prevalence and distribution of osteophytes and bone erosions by ultrasound

Among 188 PsA patients, ultrasound detected osteophytes in 41.5% (PIP joints), 32.5% (DIP joints), 6.4% (MCP joints) and 2.7% (wrists) of patients, while bone erosions were found in 28.7% (MCP joints), 28.2% (PIP joints), 23.9% (wrists) and 16.5% (DIP joints) of patients. Among the different joint regions, the PIP and DIP joints had the highest prevalence of osteophytes.

## 4 Discussion

This study offers a comprehensive assessment of the concordance between PE findings and ultrasound detection of inflammatory lesions in patients with PsA, focusing on the wrists and finger joints. The findings demonstrate significant discrepancies between PE and US-detected inflammation, highlighting the heterogeneous nature of PsA across different joints and lesion types (23). Ultrasound has been demonstrated to be a highly sensitive technique, especially when compared to PE, for assessing both the activity and damage of peripheral joints in PsA (24, 25).

The overall moderate concordance between PE and US findings observed in this study is consistent with previous research in PsA populations (25–27). Specifically, this study revealed that joint swelling had a higher concordance and correlation with US-detected inflammation compared to tenderness. Our data demonstrated that ultrasound identified inflammatory lesions in 32.6% of swollen joints without tenderness, compared to only 14.9% in tender joints without swelling. This finding aligns with previous evidence suggesting that joint swelling is more indicative of underlying inflammation (26, 27). This observation highlights the limitations of relying solely on tenderness which can be influenced by various factors such as pain sensitivity, mechanical irritation, or osteoarthritis, and may not always reflect active inflammation (26, 28, 29). Therefore, in patients with PsA, tenderness is not as specific as swelling in evaluating the inflammation of joints and periarticular structures when compared to swelling.

The study reveals important variations in concordance patterns across different joint types and specific lesion types. In the MCP and wrist joints, synovitis displayed a higher level of concordance with clinical findings compared to tenosynovitis/paratenonitis. This suggests that clinical examination may be more adept at detecting synovitis in these particular areas. On the other hand, tenosynovitis/paratenonitis exhibited better concordance with PE findings in most PIP joints, suggesting that this type of lesion may be more pronounced and therefore easier to detect in PIP joints.

Enthesitis, especially in DIP joints, demonstrated a higher concordance with clinical findings than other lesion types. This is consistent with its characteristic prominence in PsA. Since enthesitis of the hands is typically observed at the level of the distal attachment of the extensor and flexor tendons, the concordance between enthesitis and PE was calculated at the PIP1 and DIP2–5 joints. The standardized OMERACT criteria for enthesitis detected by US have probably played a crucial role in improving the reliability of detection of this feature, especially in DIP involvement (22).

One of the key findings of this study is the ability of ultrasound to detect subclinical inflammation, particularly in complex articular regions (27). Notably, the wrist had more PE-/US+ joints than other joint regions, probably due to its anatomical complexity. As a multi-compartment joint with interconnected bones sharing a common synovial cavity, its thin dorsal capsule and synovial folds make clinical swelling difficult to detect, especially in early disease or mild inflammation. This highlights the superior sensitivity of ultrasound in detecting subclinical inflammation, which is consistent with previous studies (25, 30, 31). Subclinical inflammation is of particular concern, as it may contribute to long-term joint damage if left untreated (32). Recent research has highlighted that subclinical dactylitis, although not detectable on PE, also represents a more active and severe phenotype of PsA (33). Dactylitis often involves multiple structures, making US an invaluable tool for capturing the full extent of inflammatory activity and guiding more aggressive treatment strategies (34). However, in our cases of dactylitis (with predominant toe involvement), US detected inflammatory lesions in only 66.7% of clinically affected fingers, demonstrating equivalent frequencies of synovitis and tenosynovitis/paratenonitis. Our findings may underestimate the true inflammatory burden in dactylitis, as emerging evidence indicates this condition involves additional pathological features (e.g., fibrous pulley inflammation and periosteal involvement) not currently captured by the Dactylitis Global Sonographic (DACTOS) score (35, 36). Future studies should adopt expanded scanning protocols to improve sensitivity and reflect the multi-structural pathology of dactylitis.

This study further highlights the discrepancy between clinical and US findings in PsA. Previous research has similarly demonstrated such discrepancies, where PE often underestimates the extent of inflammation, particularly in early PsA or in joints that appear clinically inactive (37). In MCP joints, swelling is more difficult to detect due to their deeper anatomical positioning and thicker surrounding tissues. This anatomical difference explains why tenderness in MCP3–5 joints correlated better with US findings than swelling, whereas interphalangeal joints, with their thinner capsules, allow for more accurate clinical detection of swelling and tenderness. Additionally, the higher prevalence of tenderness or swelling in DIP and PIP joints without US-detected inflammation may reflect their superficial anatomy, increased mechanical stress, and the potential for non-inflammatory conditions, such as osteophytes, that may mimic inflammatory signs (38). In our study, we found that osteophytes were more frequently detected in PIP and DIP joints than in MCP joints or wrists, which is consistent with previous findings and may contribute to the clinical-US discrepancies in these areas (20). These discrepancies highlight the importance for clinicians to integrate US into routine practice to improve diagnostic accuracy and better tailor treatment strategies (39).

The results of this study reinforce the growing recognition that clinical examination alone may not fully capture the extent of inflammation in PsA (26, 30, 31). Our analyses demonstrated significantly stronger correlations between ultrasound composite scores and objective markers of inflammation compared to subjective clinical assessments, establishing US as a more reliable and objective modality for evaluating true inflammatory burden. These findings underscore the complementary diagnostic role of US in the assessment of hand and wrist involvement in PsA, highlighting three key clinical applications: sensitive detection of subclinical inflammation, particularly in deep joints such as the wrist; differentiation of true inflammatory changes from osteophyte mimicking lesions in the PIP/DIP joints; and identification of enthesitis at DIP joints to inform precision therapy selection. As recommended by EULAR, incorporating US into routine clinical assessment may also prevent unnecessary interventions or overtreatment due to misinterpreted clinical signs (4). Although this study provides valuable insights, there are several major limitations that should be acknowledged. First, the cross-sectional design precludes assessment of temporal changes in clinical-US correlations or therapeutic responses; therefore, longitudinal studies are therefore needed to evaluate the evolution of imaging findings with disease progression. Second, the sonographers were not blinded to the presence of psoriatic plaques near the scanned joints, possibly leading to bias in interpreting pathology. Third, we did not include foot and ankle joints in our study, despite their frequent involvement in PsA. Fourth, enthesitis at the central slip insertion at the level of the PIP joint was not assessed, which may underestimate the true prevalence of enthesitis in PsA. Future studies should include this site for a more comprehensive assessment. Fifth, the composite ultrasound sum score requires external validation, and its weighting methodology and derivation algorithm need further investigation in future studies. Minor limitations include incomplete dactylitis assessment (missing pulley/periosteal assessment), patient-level rather than joint-level osteophyte analysis, and undifferentiated synovitis subtypes with potentially distinct clinical implications (40).

Future research directions should address: whether US-guided treatment strategies yield superior clinical outcomes compared to conventional assessment; the development of comprehensive, standardized US evaluation protocols encompassing all relevant joints and pathological features; and the optimal integration of US findings with clinical assessment in therapeutic decision-making.

## 5 Conclusion

In conclusion, this study demonstrates moderate concordance but significant variation between clinical and sonographic findings in PsA across different joints and lesions. Ultrasound demonstrated superior sensitivity for detecting subclinical inflammation and differentiating inflammatory from structural pathology. These findings strongly support the integration of ultrasound into routine PsA management to enhance diagnostic accuracy. Future longitudinal studies should further evaluate the role of ultrasound in tracking disease progression and long-term outcomes to optimize precision medicine approaches in PsA.

## Data Availability

The original contributions presented in the study are included in the article/**Supplementary Material**. Further inquiries can be directed to the corresponding author.

## References

[1] FitzGerald O Ogdie A Chandran V Coates LC Kavanaugh A Tillett W. Psoriatic arthritis. Nature reviews Disease primers. (2021) 7. doi: 10.1038/s41572-021-00293-y 34385474

[2] Gladman DD Antoni C Mease P Clegg DO Nash P. Psoriatic arthritis: epidemiology, clinical features, course, and outcome. Ann Rheum Dis. (2005) 64 Suppl 2:ii14–7. doi: 10.1136/ard.2004.032482 PMC1766874 15708927

[3] Zabotti A Bandinelli F Batticciotto A Scirè CA Iagnocco A Sakellariou G. Musculoskeletal ultrasonography for psoriatic arthritis and psoriasis patients: A systematic literature review. Rheumatol (Oxford England). (2017) 56:1518–32. doi: 10.1093/rheumatology/kex179 28521047

[4] Mandl P Navarro-Compán V Terslev L Aegerter P van der Heijde D D’Agostino MA. Eular recommendations for the use of imaging in the diagnosis and management of spondyloarthritis in clinical practice. Ann Rheum Dis. (2015) 74:1327–39. doi: 10.1136/annrheumdis-2014-206971 25837448

[5] Zabotti A Salvin S Quartuccio L De Vita S. Differentiation between early rheumatoid and early psoriatic arthritis by the ultrasonographic study of the synovio-entheseal complex of the small joints of the hands. Clin Exp Rheumatol. (2016) 34:459–65. 26939710

[6] Sapundzhieva T Karalilova R Batalov A. Hand ultrasound patterns in rheumatoid and psoriatic arthritis: the role of ultrasound in the differential diagnosis. Rheumatol Int. (2020) 40:837–48. doi: 10.1007/s00296-020-04559-8 32211929

[7] Hammer HB Michelsen B Sexton J Haugen IK Provan SA Haavardsholm EA. Swollen, but not tender joints, are independently associated with ultrasound synovitis: results from a longitudinal observational study of patients with established rheumatoid arthritis. Ann Rheum Dis. (2019) 78:1179–85. doi: 10.1136/annrheumdis-2019-215321 31171525

[8] Sun X Deng X Xie W Wang L Wang Y Zhang Z. The agreement between ultrasound-determined joint inflammation and clinical signs in patients with rheumatoid arthritis. Arthritis Res Ther. (2019) 21:100. doi: 10.1186/s13075-019-1892-0 30995934 PMC6471966

[9] Florescu A Muşetescu AE Florescu LM Bondari A Ciurea PL Vere CC. The role of ultrasound in assessing hand joints and tendons in psoriatic arthritis. Curr Health Sci J. (2019) 45:198–203. doi: 10.12865/chsj.45.02.11 31624648 PMC6778302

[10] Taylor W Gladman D Helliwell P Marchesoni A Mease P Mielants H. Classification criteria for psoriatic arthritis: development of new criteria from a large international study. Arthritis rheumatism. (2006) 54:2665–73. doi: 10.1002/art.21972 16871531

[11] Fredriksson T Pettersson U. Severe psoriasis–oral therapy with a new retinoid. Dermatologica. (1978) 157:238–44. doi: 10.1159/000250839 357213

[12] Coates LC Fransen J Helliwell PS. Defining minimal disease activity in psoriatic arthritis: A proposed objective target for treatment. Ann Rheum Dis. (2010) 69:48–53. doi: 10.1136/ard.2008.102053 19147615

[13] Schoels M Aletaha D Funovits J Kavanaugh A Baker D Smolen JS. Application of the darea/dapsa score for assessment of disease activity in psoriatic arthritis. Ann Rheum Dis. (2010) 69:1441–7. doi: 10.1136/ard.2009.122259 20525844

[14] Backhaus M Burmester GR Gerber T Grassi W Machold KP Swen WA. Guidelines for musculoskeletal ultrasound in rheumatology. Ann Rheum Dis. (2001) 60:641–9. doi: 10.1136/ard.60.7.641 PMC1753749 11406516

[15] Wakefield RJ Balint PV Szkudlarek M Filippucci E Backhaus M D’Agostino MA. Musculoskeletal ultrasound including definitions for ultrasonographic pathology. J Rheumatol. (2005) 32:2485–7. 16331793

[16] Bruyn GA Iagnocco A Naredo E Balint PV Gutierrez M Hammer HB. Omeract definitions for ultrasonographic pathologies and elementary lesions of rheumatic disorders 15 years on. J Rheumatol. (2019) 46:1388–93. doi: 10.3899/jrheum.181095 30709946

[17] Szkudlarek M Court-Payen M Jacobsen S Klarlund M Thomsen HS Østergaard M. Interobserver agreement in ultrasonography of the finger and toe joints in rheumatoid arthritis. Arthritis rheumatism. (2003) 48:955–62. doi: 10.1002/art.10877 12687537

[18] Ellegaard K Torp-Pedersen S Holm CC Danneskiold-Samsøe B Bliddal H. Ultrasound in finger joints: findings in normal subjects and pitfalls in the diagnosis of synovial disease. Ultraschall der Med (Stuttgart Germany: 1980). (2007) 28:401–8. doi: 10.1055/s-2007-963170 17680518

[19] Macía-Villa C Falcao S Gutierrez M Medina J Hammer HB De Miguel E. Peritenon extensor tendon inflammation in psoriatic arthritis is an enthesitis-related lesion. J Rheumatol. (2019) 46:1295–8. doi: 10.3899/jrheum.180856 30709962

[20] Polachek A Furer V Zureik M Nevo S Mendel L Levartovsky D. Ultrasound, magnetic resonance imaging and radiography of the finger joints in psoriatic arthritis patients. Rheumatol (Oxford England). (2022) 61:563–71. doi: 10.1093/rheumatology/keab272 33734348

[21] Acosta-Felquer ML Ruta S Rosa J Marin J Ferreyra-Garrot L Galimberti ML. Ultrasound entheseal abnormalities at the distal interphalangeal joints and clinical nail involvement in patients with psoriasis and psoriatic arthritis, supporting the nail-enthesitis theory. Semin Arthritis rheumatism. (2017) 47:338–42. doi: 10.1016/j.semarthrit.2017.05.002 28648658

[22] Balint PV Terslev L Aegerter P Bruyn GAW Chary-Valckenaere I Gandjbakhch F. Reliability of a consensus-based ultrasound definition and scoring for enthesitis in spondyloarthritis and psoriatic arthritis: an omeract us initiative. Ann Rheum Dis. (2018) 77:1730–5. doi: 10.1136/annrheumdis-2018-213609 30076154

[23] Kavanaugh A Helliwell P Ritchlin CT. Psoriatic arthritis and burden of disease: patient perspectives from the population-based multinational assessment of psoriasis and psoriatic arthritis (Mapp) survey. Rheumatol Ther. (2016) 3:91–102. doi: 10.1007/s40744-016-0029-z 27747516 PMC4999579

[24] Weiner SM Jurenz S Uhl M Lange-Nolde A Warnatz K Peter HH. Ultrasonography in the assessment of peripheral joint involvement in psoriatic arthritis: A comparison with radiography, mri and scintigraphy. Clin Rheumatol. (2008) 27:983–9. doi: 10.1007/s10067-008-0835-y 18259687

[25] Wiell C Szkudlarek M Hasselquist M Møller JM Vestergaard A Nørregaard J. Ultrasonography, magnetic resonance imaging, radiography, and clinical assessment of inflammatory and destructive changes in fingers and toes of patients with psoriatic arthritis. Arthritis Res Ther. (2007) 9:R119. doi: 10.1186/ar2327 18001463 PMC2246238

[26] Felbo SK Wiell C Østergaard M Poggenborg RP Bøyesen P Hammer HB. Do tender joints in active psoriatic arthritis reflect inflammation assessed by ultrasound and magnetic resonance imaging? Rheumatol (Oxford England). (2022) 61:723–33. doi: 10.1093/rheumatology/keab384 33895799

[27] Dubash SR Alabas OA Michelena X Garcia-Montoya L De Marco G Merashli M. Ultrasound shows swollen joints are the better proxy for synovitis than tender joints in dmard-naïve early psoriatic arthritis. Rheumatol Adv Pract. (2021) 5:rkab086. doi: 10.1093/rap/rkab086 35284780 PMC8908782

[28] Anderson KO Green CR Payne R. Racial and ethnic disparities in pain: causes and consequences of unequal care. J Pain. (2009) 10:1187–204. doi: 10.1016/j.jpain.2009.10.002 19944378

[29] Eder L Thavaneswaran A Chandran V Cook R Gladman DD. Factors explaining the discrepancy between physician and patient global assessment of joint and skin disease activity in psoriatic arthritis patients. Arthritis Care Res (Hoboken). (2015) 67:264–72. doi: 10.1002/acr.22401 25047020

[30] Zuliani F Zabotti A Errichetti E Tinazzi I Zanetti A Carrara G. Ultrasonographic detection of subclinical enthesitis and synovitis: A possible stratification of psoriatic patients without clinical musculoskeletal involvement. Clin Exp Rheumatol. (2019) 37:593–9. 30620282

[31] Norden A Oulee A Ivanic M Javadi SS Han G Wu JJ. The use of ultrasound to detect enthesitis as a potential guide for intervention in patients with psoriasis at risk of psoriatic arthritis: A systematic review. Int J Dermatol. (2023) 62:973–9. doi: 10.1111/ijd.16645 37005348

[32] Ribeiro AL Eder L. From psoriasis to psoriatic arthritis: ultrasound insights connecting psoriasis with subclinical musculoskeletal inflammation and the path to psoriatic arthritis. Curr Rheumatol Rep. (2024) 26:235–47. doi: 10.1007/s11926-024-01146-9 38512585

[33] Song Z Geng Y Zhang X Deng X Zhang Z. Subclinical dactylitis represents a more active phenotype of psoriatic arthritis. Joint Bone Spine. (2024) 92:105784. doi: 10.1016/j.jbspin.2024.105784 39326834

[34] McGonagle D Tan AL Watad A Helliwell P. Pathophysiology, assessment and treatment of psoriatic dactylitis. Nat Rev Rheumatol. (2019) 15:113–22. doi: 10.1038/s41584-018-0147-9 30610219

[35] Ricci V Tamborrini G Zunica F Chang KV Kara M Farì G. High-resolution ultrasound imaging of elementary lesions in dactylitis. J ultrasound. (2024) 27:281–90. doi: 10.1007/s40477-023-00834-z PMC11178685 38006512

[36] Zabotti A Sakellariou G Tinazzi I Idolazzi L Batticciotto A Canzoni M. Novel and reliable dactylitis global sonographic (Dactos) score in psoriatic arthritis. Ann Rheum Dis. (2020) 79:1037–43. doi: 10.1136/annrheumdis-2020-217191 32430315

[37] Husic R Gretler J Felber A Graninger WB Duftner C Hermann J. Disparity between ultrasound and clinical findings in psoriatic arthritis. Ann Rheum Dis. (2014) 73:1529–36. doi: 10.1136/annrheumdis-2012-203073 23740228

[38] Husic R Finzel S Stradner MH Dreu M Hofmeister A Beham-Schmid C. Ultrasound in osteoarthritis of the hand: A comparison to computed tomography and histology. Rheumatol (Oxford England). (2022) 61:Si73–si80. doi: 10.1093/rheumatology/keab526 34244721

[39] Gazel U Solmaz D Ayan G Ivory C Karsh J Aydin SZ. Accuracy of physical examination to detect synovial and extra-synovial pathologies in psoriatic arthritis in comparison to ultrasonography. J Clin Med. (2020) 9. doi: 10.3390/jcm9092929 PMC7564699 32927920

[40] Ricci V Ricci C Tamborrini G Chang KV Mezian K Zunica F. From histology to sonography in synovitis: euro-musculus/usprm approach. Pathol Res Pract. (2023) 241:154273. doi: 10.1016/j.prp.2022.154273 36563558

