# Supplementary material for: The concordance of swelling/tenderness with ultrasound-detected inflammatory lesions in patients with psoriatic arthritis
Source: Front Immunol. 2025 May 30;16:1562996. doi: 10.3389/fimmu.2025.1562996 (PMC12162965; doi:10.3389/fimmu.2025.1562996)
Supplement: Supplementary file 1 [file Table1.docx]

**Supplementary Table 1** Association between clinical findings and ultrasound sum score of wrists and hands

|  | TJC(68) | SJC(66) | Ptpain | PGA | EGA | HAQ | ESR | CRP | PASI | DAPSA |
| --- | --- | --- | --- | --- | --- | --- | --- | --- | --- | --- |
| A global ultrasound sum score of wrists and hands | 0.281^**^ | 0.488^**^ | 0.183^*^ | 0.170^*^ | 0.307^**^ | 0.155^*^ | 0.240^**^ | 0.392^**^ | -0.098 | 0.406^**^ |

Correlation coefficients (ρ) according to Spearman’s rank correlation test are shown.

CRP, C-reactive protein; DAPSA, disease activity index for psoriatic arthritis; EGA, evaluator’s global assessment of disease activity; ESR, erythrocyte sedimentation rate; HAQ, Health Assessment Questionnaire; PASI, psoriasis area and severity index; PGA, patient’s global assessment of disease activity; Ptpain, patients’ pain assessment; SJC, swollen joint count; TJC, tender joint count.

*, P-value＜0.05, **, P-value＜0.01.
